# Complete Genome Sequence of Coprobacter fastidiosus JCM 33896^T^

**DOI:** 10.1128/mra.00326-23

**Published:** 2023-06-05

**Authors:** Kazuyoshi Koike, Dieter M. Tourlousse, Mayu Hamajima, Yuji Sekiguchi

**Affiliations:** a Biomedical Research Institute, National Institute of Advanced Industrial Science and Technology (AIST), Tsukuba, Ibaraki, Japan; Loyola University Chicago

## Abstract

We generated a complete genome sequence of Coprobacter fastidiosus JCM 33896^T^ by nanopore sequencing. The genome consists of a single circular chromosome of 3,444,538 bp with a G+C content of 38.4%. Annotation predicted 15 rRNA genes, 67 tRNA genes and 2,662 protein-coding sequences.

## ANNOUNCEMENT

Coprobacter fastidiosus is an obligatory anaerobic Gram-negative bacterium that was originally isolated from feces of a healthy infant (1) and represents the first of two validly described species within the genus *Coprobacter* (2). Members of the species are frequently detected in human feces and have been associated with a range of health conditions; for example, *C. fastidiosus*, as well as Coprobacter secundus, was found to be less abundant in patients with chronic obstructive pulmonary disease (3), and the abundance of members of *Coprobacter* has been associated with ulcerative colitis (4). In another study, the abundance of *Coprobacter* was increased in feces of patients with neurosyphilis (5), suggesting a potential association with neuropsychiatric disorders. In this work, we generated a complete genome sequence of the authentic type strain of *C. fastidiosus* (JCM 33896^T^ = DSM 26242^T^ = VKM B-2743^T^) by Oxford Nanopore Technologies (ONT) sequencing.

Cells were obtained from the Japan Collection of Microorganisms (JCM) and cultured in peptone-yeast extract-glucose (PYG) medium under an atmosphere of N_2_:CO_2_ (80:20, vol/vol) at 37°C. Genomic DNA was extracted using the MagAttract high-molecular-weight (HMW) DNA kit (Qiagen). Libraries for sequencing were constructed with ONT’s Native Barcoding kit 24 (SQK-NBD114.24), and sequenced on an R10.4.1 flow cell with a GridION device, with default settings (translocation speed of 400 basepairs/s and sampling rate of 4 kHz). All bioinformatics tools were run with default parameters, unless stated otherwise. Basecalling was performed using Dorado v0.2.1 (https://github.com/nanoporetech/dorado) with model dna_r10.4.1_e8.2_400bps_sup@v4.0.0. Duplex Tools v0.3.1 (https://github.com/nanoporetech/duplex-tools), four iterations with option -allow_multiple_splits, was used to identify and split the chimeric and/or concatenated reads. Guppy’s guppy_barcoder command (v6.4.6) with concurrent barcode trimming (options -enable_trim_barcodes -barcode_kits SQK-NBD114-24) was then used for demultiplexing libraries. After removal of reads with a length of <1,000 bases and an average quality score of <12 using NanoFilt v2.8.0 (6), a set of high-quality reads was obtained using Filtlong v0.2.0 (https://github.com/rrwick/Filtlong), with option -mean_q_weight 10. This resulted in a total of 172,610 reads (516,695,412 bases with an estimated genome coverage of 150×) with an *N*_50_ of 3,501 bases. The genome was assembled using Flye v2.9.1 (7), followed by polishing by Medaka v1.7.3 (https://github.com/nanoporetech/medaka) with model r1041_e82_400bps_sup_v4.0.0. Completeness (99.6%) and contamination (0.0%) of the genome were confirmed using CheckM v1.1.3, lineage_wf (8). The genome was annotated with the DDBJ Fast Annotation and Submission Tool v1.2.18 (9).

The genome of *C. fastidiosus* JCM 33896^T^ consists of a single circular chromosome with a length of 3,444,538 bp. It has a G+C content of 38.4% and was predicted to contain 5 complete rRNA operons and 67 tRNA genes and to encode 2,662 proteins. The availability of a complete genome of *C. fastidiosus* JCM 33896^T^ will contribute to improving our understanding of this bacterium in the human gut.

### Data availability.

This genome sequence has been deposited in DDBJ/EMBL/GenBank under the accession number AP028032. Sequencing reads are available in the DDBJ Sequence Read Archive under accession number DRR457763.
